# Further In Vitro and Ex Vivo Pharmacological and Kinetic Characterizations of CCF219B: A Positive Allosteric Modulator of the α_1A_-Adrenergic Receptor

**DOI:** 10.3390/ph18040476

**Published:** 2025-03-27

**Authors:** Robert S. Papay, Dianne M. Perez

**Affiliations:** The Department of Cardiovascular & Metabolic Sciences, Lerner Research Institute, The Cleveland Clinic Foundation, 9500 Euclid Ave, Cleveland, OH 44195, USA

**Keywords:** Alzheimer’s disease, adrenergic receptor, GPCR, allosteric modulator, kinetics

## Abstract

**Background:** Alterations in the adrenergic system have been associated with the pathophysiology of Alzheimer’s disease (AD). A novel α_1A_-adrenergic receptor (AR)-positive allosteric modulator (PAM), CCF219B, has been shown to outperform donepezil with rescue of AD cognition/memory deficits with a reduction in amyloid biomarkers and without cardiovascular side effects. Initial pharmacological analysis in transfected cell lines revealed a signal bias with increased efficacy (but not potency) of cAMP signaling and ligand selectivity for norepinephrine (NE). As most GPCR allosteric modulators change the potency of agonists, we hypothesized and now report that CCF219B induced additional aspects of its allosteric interactions with NE that may provide mechanistic insight. **Methods:** Using Rat-1 fibroblasts stably transfected with α_1A_-AR, we determined the activation profile of pERK and p38 messengers by CCF219B in the presence of NE. Using membranes prepared from the stably transfected fibroblasts or from the brain of WT mice or the AD mouse model, hAPP(lon), equilibrium or kinetic radioligand-binding analyses were performed. **Results:** We identified p-ERK1/2 but not p38 as an additional signal pathway that is potentiated by CCF219B in the presence of NE. An analysis of binding studies of CCF219B in membranes derived from the brains of WT or hAPP(lon) mice revealed profiles that were time-dependent and resulted in an increase in α_1A_-AR expression that was unaltered in the presence of cycloheximide or when performed at 37 °C. hAPP(lon) mice displayed a reduction in α_1A_-AR-binding sites that were rescued upon prolonged incubation with CCF219B but also displayed a compensatory increase in α_1B/D_-AR subtype expression. Binding kinetics reveal that CCF219B can decrease the association rate of ^3^H-NE but only in the presence of GTP. The association rate increased for the radiolabeled antagonist, ^125^I-HEAT. There were no changes in the dissociation rate of either radiolabel. **Conclusions:** CCF219B affects the association but not the dissociation rate of NE and explains its ability to increase the active state of the receptor by promoting a pre-coupled conformation, consistent with increasing efficacy but not potency. Potentiation of pERK may contribute to CCF219B’s ability to confer neuroprotection and be pro-cognitive in AD. CCF219B’s ability to increase the expression of α_1A_-AR provides a positive feedback loop and strengthens the hypothesis that α_1_-AR subtypes may be involved in AD etiology and/or progression.

## 1. Introduction

α_1_-Adrenergic receptors (ARs) regulate the function of the sympathetic nervous system, along with α_2_- and β-ARs, through the binding of norepinephrine (NE) and epinephrine. While the three subtypes (i.e., α_1A_, α_1B_, and α_1D_) have been best studied for their cardiovascular regulation, α_1A_-ARs hold promise to treat Alzheimer’s disease (AD) with their neurogenic, neuroprotective, and pro-cognitive/memory profile [1].

NE’s involvement in AD is associated with decreased levels and cognitive deficits [2,3], decreased amyloid clearance [4], and possible etiology [5,6]. NE activation improves memory and cognition [7]. The use of genetically modified mouse models confirmed the α_1A_-AR subtype in mediating NE’s effects on learning and memory functions, and synaptic plasticity in the hippocampus [8]. In humans, there is loss of α_1A_-AR mRNA in the hippocampus of AD patients [9], and a polymorphism of α_1A_-AR is associated with AD progression [10].

We recently developed and characterized the first positive allosteric modulator (PAM) of α_1A_-AR [11] that can reverse AD deficits in long-term potentiation and cognitive behaviors in AD mouse models with almost total clearance of amyloids [12], suggesting a reversal of AD progression. There was no effect on blood pressure, a common side effect of α_1_-AR activation, because of the signal bias of CCF219B, which enhances cognitive/memory cAMP signals but not vascular-contracting inositol phosphates that increase calcium signaling [12]. In addition, CCF219B is a pure PAM, having no basal signal transduction [11] and only acting when NE is present. These properties may also reduce AD risk and pathology, which are linked to high blood pressure [13].

We now report that CCF219B also potentiates extracellular signal-regulated kinase (ERK)-mediated signaling, known for its neuro- and cardioprotective functions [14,15,16,17]. We also expand upon CCF219B’s pharmacological characterization to show the effects on binding kinetics that confirm its allosteric mechanism. Finally, we confirm the involvement of the α_1A_-AR subtype in AD by demonstrating decreased expression in the human amyloid precursor protein (hAPP) AD mouse model and reversal of this deficit by prolonged incubation with CCF219B, which modulates α_1A_-AR expression levels in a positive feedback loop.

## 2. Results

**Figure 1:**
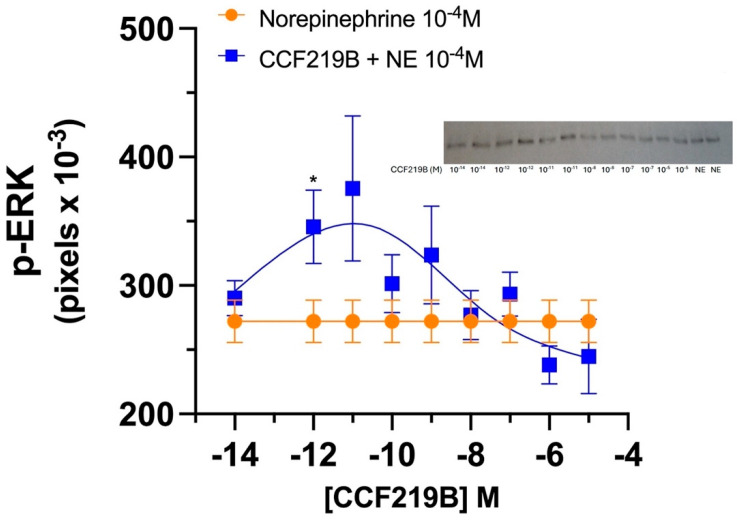
PAM assay of p-ERK1/2. Rat-1 fibroblasts expressing α_1A_-AR were incubated with either NE alone (10^−4^ M) (orange circles) or in the presence of CCF219B (10^−8^ M) (blue squares). The addition of CCF219B produced a potentiation of the p-ERK signaling over that of NE alone. N = 6 independent experiments performed in duplicate. Data were fitted to a bell-shaped curve according to the equation in GraphPad Prism (Version 10.2.0) *p* < 0.036, and using the Mann–Whitney test for significance; * *p* < 0.039, unpaired student’s *t*-test.

After the demonstration that CCF219B is both ligand- and signal-biased and can potentiate the NE-mediated cAMP response without effects on inositol phosphate signaling [11]), we next determined whether CCF219B can affect the mitogen-activated protein kinase (MAPK) signaling of NE. α_1_-ARs have previously been shown to couple to p-ERK and p-38 signal transductions [18]. After performing time-course studies (Figure S1), it was determined that CCF219B could affect NE-mediated phosphorylation of ERK but not p38. We next determined the efficacy and potency of CCF219B to evoke the p-ERK signal by performing a PAM assay, for which the concentration of CCF219B was varied while NE was held constant at 10^−4^ M. We found that CCF219B produced a significant increase in efficacy (30% increase, *p* < 0.036) with a potency (sub nM) (Figure 1) similar to the NE-mediated cAMP response [12].

**Figure 2:**
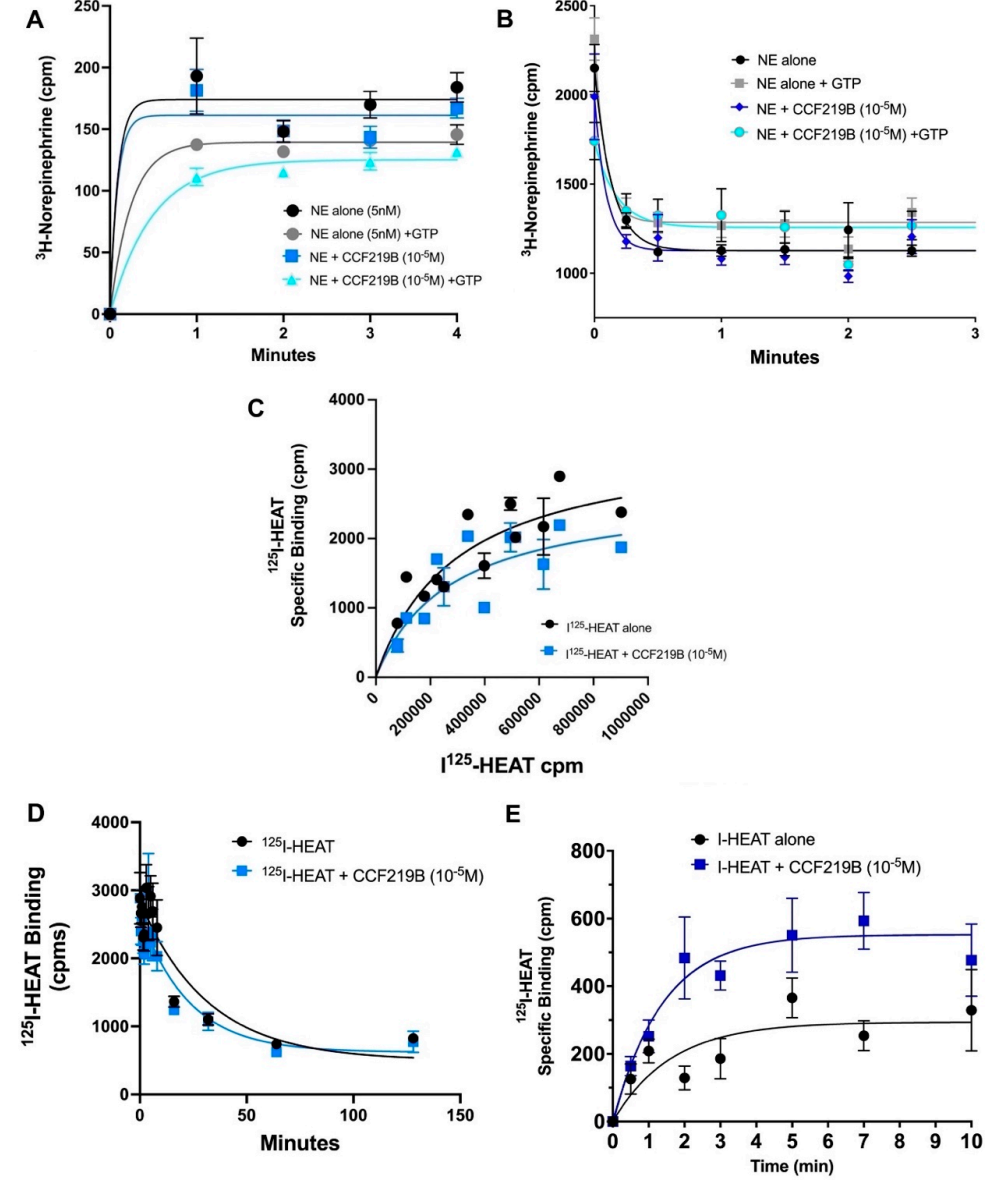
Kinetic analysis of CCF219B (10^−5^ M) binding to ^3^H-NE (5 nM) or ^125^I-HEAT (40 pM) in stably transfected α_1A_-AR or WT mouse brain membranes. All incubations included the β-AR blocker propranolol (10 μM) and the α_2_-AR blocker rawoulscine (10 μM). Data were fitted using the equation in GraphPad Prism for association kinetics—one conc. of hot. (**A**) Association curves of ^3^H-NE with or without GTP and/or CCF219B in α_1A_-AR membranes. N = 6 independent experiments performed in duplicate. NE + CCF219B (10^−5^ M) + GTP was statistically significant from NE + CCF219B (10^−5^ M). *p* < 0.0002 (ANOVA; Tukey’s post test). (**B**) Dissociation curves of ^3^H-NE with or without GTP and/or CCF219B in α_1A_-AR membranes. N = 5 independent experiments performed in duplicate. Data were fitted to the equation in GraphPad Prism using dissociation—two-phase exponential decay. (**C**) Equilibrium saturation studies of ^125^I-HEAT with or without CCF219B in α_1A_-AR membranes. N = 3 independent experiments performed in duplicate. (**D**) Dissociation curves of ^125^I-HEAT with or without CCF219B in α_1A_-AR membranes. N = 3 independent experiments performed in duplicate. Data were fitted using the dissociation two-phase exponential decay in GraphPad Prism. (**E**) Association curves of ^125^I-HEAT with or without CCF219B in WT mouse brain membranes. The addition of CCF219B was statistically significant (*p* < 0.01) (paired *t*-test; two-tailed; r = 0.78). N = 3 independent experiments performed in duplicate.

We next performed kinetic radioligand-binding studies in α_1A_-AR-transfected fibroblasts or WT mouse brain membranes to determine whether CCF219B could affect the binding characteristics of NE, since CCF219B displayed allosteric effects against this agonist [12]. These experiments were difficult to perform because of the low affinity of NE, and the resulting fast on and off rates, resulting in limited timepoints within the first minute. Using an ice-bath (1 °C) to lower the rates of association and using guanosine triphosphate (GTP) to induce activation of the receptor/G-protein complex, we found that CCF219B (10^−5^ M) alone did not cause a change in the association rate of NE at α_1A_-AR (Figure 2A). However, there was a significant effect of CCF219B on reducing the association rate of NE to α_1A_-AR when GTP was present. ^3^H-NE (5 nM) + GTP (5 mM) resulted in a measurable k_obs_ = 4.7 ± 0.72 × 10^8^/M/min, similar to that reported for epinephrine at the β-AR [19] and for other β-AR agonists, such as isoproterenol (1–5 × 10^8^/M/min) [20], which are similar in structure and affinity to NE. Interestingly, when combined together, GTP and CCF219B reduced the association rate of NE even further by eight-fold to k_obs_ = 6 + 0.85 × 10^7^/M/min (*p* < 0.0002). There was no significant changes in the dissociation rate of ^3^H-NE when CCF219B (10^−5^ M) was added. While the rate of ^3^H-NE dissociation decreased when GTP was applied, there were no changes when performed in the presence of CCF219B + GTP (Figure 2B).

We next performed saturation binding to determine whether CCF219B could alter the equilibrium binding of the common radiolabel ^125^I-HEAT, an α_1_-AR antagonist, as allosteric modulators can be ligand-specific in its effects. We found that CCF219B can produce a small rightward shift in the saturation curve, resulting in the lowering of the affinity (i.e., increasing K_D_) (Figure 2C). There were also minor effects from CCF219B on ^125^I-HEAT dissociation. CCF219B appeared to slightly increase the KFast dissociation rate of ^125^I-HEAT (K_off_ = 0.031 to 0.047 min^−1^) (*p* < 0.1) (Figure 2D). While the effects of CCF219B were also minor on altering ^125^I-HEAT association in transfected α_1A_-AR membranes (Figure S2), the effect became significant (*p* < 0.03) in WT mouse brain membranes, whereby CCF219B increased the association rate of ^125^I-HEAT (K_on_ = 1.5 ± 0.06 × 10^8^ M/min) by 20% to (K_on_ = 1.8 ± 0.01 × 10^8^ M/min) (*p* < 0.01) (Figure 2E).

To explore ex vivo pharmacology, we next utilized brains from both WT and hAPP-transgenic (Tg) mice similar in age (8–9 months) to the mice we used to characterize the in vivo effects of CCF219B in AD mouse models [12]. hAPP-Tg has a point mutation in the human amyloid precursor protein (hAPP) at valine 717 to isoleucine and was first identified in an English family; hence, it is called the London mutation (lon) [21] and has been shown to affect APP processing [22]. The hAPP-Tg AD mouse model displays deficits in long-term potentiation as early as 6–8 months of age [23].

**Figure 3:**
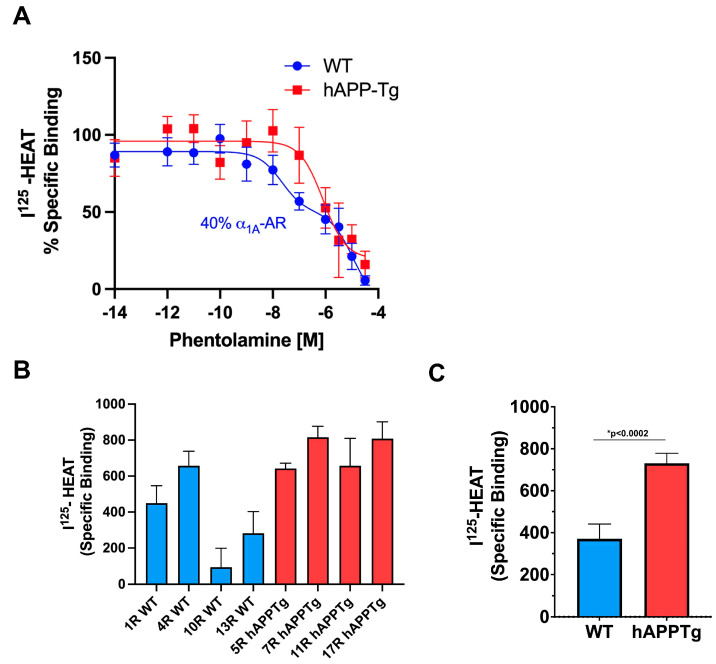
Ex vivo binding analysis of the α_1_-AR subtype distribution and the effects of CCF219B in WT or AD brain membranes. (**A**) α_1_-AR subtype analysis in WT (blue circles) and hAPP-Tg (red squares) brain membranes using phentolamine to compete with ^125^I-HEAT in the presence of β- and α_2_-AR blockers. WT membranes expressed 40% of the high-affinity α_1A_-AR subtype compared to the hAPP-Tg membranes, which fit best to a one-site log IC_50_ model of low-affinity α_1B/D_-ARs (*p* < 0.05) using the equations in GraphPad Prism (R^2^ = 0.35 versus R^2^ = 0). WT membranes fit best using the two-site log IC_50_ model (R^2^ = 0.52 versus R^2^ = 0.48). N = 14 independent experiments performed in duplicate. (**B**) Individual brains from WT (blue bars) and hAPP-Tg mice (red bars) were analyzed for the total α_1_-AR density using a saturating amount of ^125^I-HEAT (8.5 × 10^5^ cpms). N = 4 independent experiments performed in duplicate. (**C**) Statistical analysis of the data in (**B**) using an unpaired, two-tailed *t*-test, *p* < 0.0002.

In radioligand-binding studies to characterize the levels of α_1_-AR subtypes in the hAPP-Tg brains, we utilized phentolamine which has a 10–100 fold higher affinity for α_1A_-AR compared with the α_1B_- or α_1D_-AR subtypes [24] resulting in typical 2-site competition curves in tissue studies which have a mixed population of α_1_-ARs. While hAPP-Tg brain membranes were best fit to a single low-affinity site (100% α_1B/D_-ARs; IC_50_ = −6), WT brain membranes were best fit to a two-site model, representing the higher affinity α_1A_-AR (at 40%) (log IC_50_ = −7.7 ± 0.006) and a lower affinity site composed of both the α_1B_- and α_1D_-AR subtypes (log IC_50_ = −6 ± 0.33) (Figure 3A).

We then determined the total density of the α_1_-AR subtypes in individual WT and hAPP-Tg mouse brains using a saturating amount of the radioligand, ^125^I-HEAT. Analyzing the same side of the brain (right, R), three out of four individual WT brains displayed a lower total α_1_-AR density compared to the four individual hAPP-Tg brains (Figure 3B), resulting in a high statistical difference (*p* < 0.0002) (Figure 3C).

**Figure 4:**
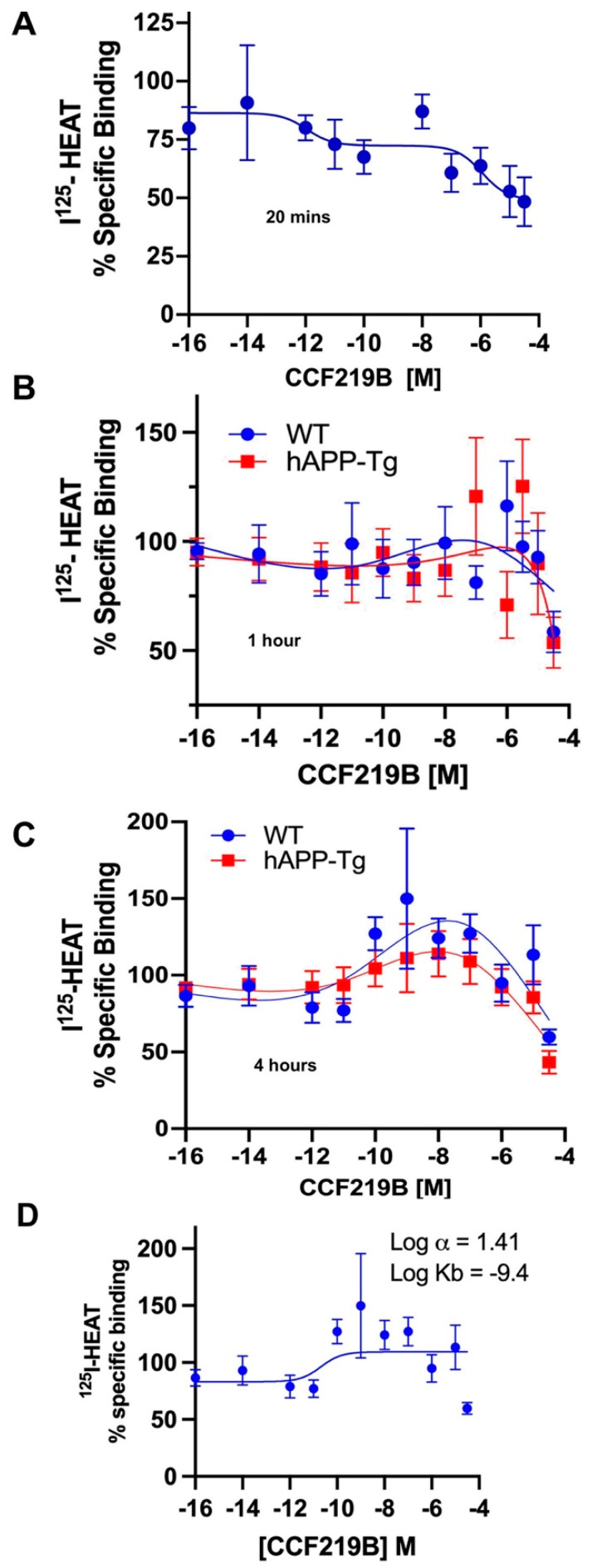
CCF219B increases expression of ^125^I-HEAT-binding sites in WT or AD brain membranes after prolonged incubation. (**A**) Binding of CCF219B versus ^125^I-HEAT after 20 min of incubation using WT brain membranes. N = 5 independent experiments performed in duplicate. A two-site inhibition curve of both high-affinity and low-affinity sites using the two-site log IC_50_ equation in GraphPad Prism. Binding of CCF219B versus ^125^I-HEAT after (**B**) 1 h or (**C**) 4 h of incubation using WT (blue circles) or hAPP-Tg (red squares) brain membranes. N = 7 independent experiments performed in duplicate. Curves were fitted through the data points using a spline- or bell-shaped equations in GraphPad Prism (**D**). WT data in (**C**) were fitted to a model of allosteric modulator binding according to the following equation in GraphPad Prism: Y = (Y0/HotOccupancy) × (RadioligandNM/(RadioligandNM + KAppNM). The analysis indicates a log α = 1.41 and log Kb = −9.4.

**Figure 5:**
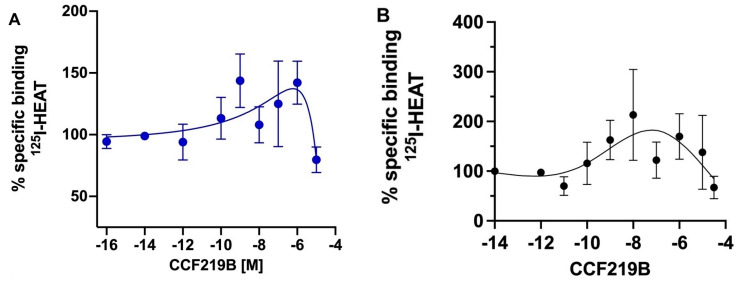
CCF219B-mediated increase in expression of α_1_-ARs is not sensitive to elevated temperature or cycloheximide. (**A**) Binding analysis of CCF219B versus ^125^I-HEAT after 4 h of incubation at 37 °C using WT brain membranes. There was no change in the binding profile compared to that in Figure 4C at 25 °C. N = 3 independent experiments performed in duplicate. (**B**) Binding analysis of CCF219B versus ^125^I-HEAT after 5 h of incubation in the presence of 25 μg/mL cycloheximide using WT brain membranes. There was no change in the binding profile compared to that in Figure 4C without the addition of cycloheximide. N = 3 independent experiments performed in duplicate. Curves were fitted through the data points using a spline- or bell-shaped equation in GraphPad Prism.

We next determined the binding profile of CCF219B in the WT and hAPP-Tg brains. Utilizing radioligand-binding studies with a series of CCF219B concentrations, a variable binding profile was apparent with differences that were dependent upon the time of incubation. Previous studies established that the time to reach equilibrium is 20–30 min; however, allosteric interactions can take longer to fully equilibrate, and allowing longer times to reach equilibrium is often recommended [25]. After a 20 min incubation, CCF219B displayed a two-site binding profile (high affinity IC_50H_ = −12 ± 3; low affinity IC_50L_ = −6 ± 1.1) in WT brain membranes similar to that published for α_1A_-AR-transfected fibroblasts [11] (Figure 4A). After 1 h of incubation, there was an increase in ^125^I-HEAT-binding sites at concentrations above 10^−10^ M of CCF219B (Figure 4B). After 4 h of incubation, the increase in ^125^I-HEAT-binding sites was greater, resulting in a profile that peaked at nM concentrations of CCF219B (Figure 4C). Curve fitting the data according to the allosteric modulator equation Y = (Y0/HotOccupancy) × (RadioligandNM/(RadioligandNM + KAppNM)), where App is the allosteric modulator and Y0 is the radioligand binding in the absence of a modulator, in GraphPad Prism calculates a log α of 1.41 and a log Kb of −9.4, consistent with a PAM and its in vivo potency. As we had never previously analyzed the competitive binding of CCF219B versus ^125^I-HEAT after 4 h of incubation in the α_1A_-AR stably transfected Rat-1 fibroblasts, we performed that study and found that the increase in ^125^I-HEAT-binding sites was apparent but not as robust as in ex vivo tissue but did mask the high affinity site for CCF219B (Figure S3). As we know that CCF219B does not increase the Bmax of ^125^I-HEAT (Figure 2B), the increase in binding could possibly be due to interactions with the membrane environment or increased stability of the receptor–ligand complex. To test this hypothesis, we performed a 4 h binding experiment at 37 °C. Increasing the temperature during ligand binding may alter the membrane’s fluidity or destabilize protein conformations/stability, which may affect the binding affinity/kinetics [26], particularly for allosteric interactions which are conformationally driven [27]. However, the increase in ^125^I-HEAT binding maintained a similar profile even after 4 h of incubation at an elevated temperature (37 °C) (Figure 5A), with the increase in binding typically beginning near 1 × 10^−10^ M of CCF219B. We also tested the effect of 25 μg/mL of cycloheximide, which can inhibit protein synthesis with the increase in ^125^I-HEAT-binding sites, but this was also without effect (Figure 5B).

**Figure 6:**
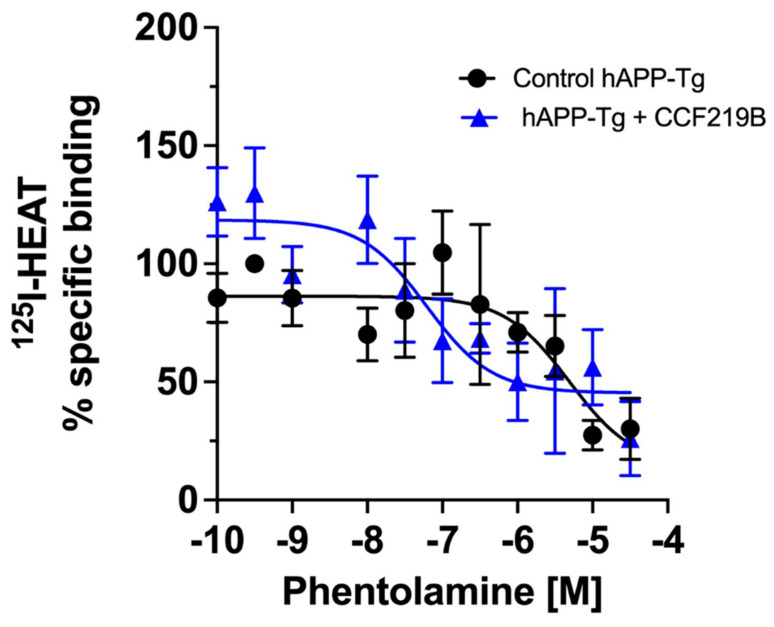
Incubation of CCF219B (10^−8^ M) rescues decreased α_1A_-AR expression in hAPP-Tg mice. Binding of phentolamine versus ^125^I-HEAT in hAPP-Tg brain membranes, which were pre-incubated for 4–5 h with (blue squares) or without (control; black circles) CCF219B (10^−8^ M). Membranes were then washed to remove CCF219B before the initiation of the binding experiment. Curves were fitted for one-site versus two-site log IC_50_ equations in GraphPad Prism. The % specific binding was normalized to the control, which displayed a one-site model with a single low-affinity site for phentolamine (i.e., 100% α_1B/D_-ARs; IC_50_ = −5.3). In the presence of CCF219B, the % specific binding increased and there was an increase in the amount of α_1A_-ARs with one-site, high affinity binding for phentolamine (i.e., 100% α_1A_-AR; IC_50_ = −7.2). N = 5 independent experiments performed in duplicate.

As ^125^I-HEAT is a specific radioligand of the α_1_-ARs subtypes, with the inclusion of both β- and α_2_-AR blockers in the binding experiments, the increase in binding we observed was likely due to increased α_1_-AR expression. To confirm this and to determine which α_1_-AR subtype is being upregulated, we pre-incubated brain membranes from the hAPP-Tg mice with CCF219B (10^−8^ M) or HEM buffer (control) for 4–5 h. The membranes were then washed to remove all traces of CCF219B, which would interact with the radioligand, and then subjected to binding studies using phentolamine to determine the percentage of high- versus low-affinity sites. We found that control hAPP-Tg membranes displayed a similar one-site model (100%) of low-affinity (α_1B_ and α_1D_) α_1_-ARs, similar as shown in Figure 3A (IC_50_ = −5.3 ± 0.57) (Figure 6). However, upon 4–5 h of pre-incubation with CCF219B (10^−8^ M), the amount of ^125^I-HEAT-binding sites increased as expected but was composed mainly (one-site model) of the high-affinity α_1A_-AR (IC_50_ = −7.2 ± 0.4) (Figure 6).

## 3. Discussion

Acetylcholinesterase inhibitors, such as donepezil, are approved drugs for treating AD but result in severe side effects and have limited and short-acting efficacy that fails to arrest the progression of the disease. CCF219B holds promise as an alternative treatment option that improves long-term potentiation and decreases β-amyloid levels better than donepezil, with no detectable side effects, particularly on blood pressure [12]. α_1A_-AR activators also confer cardioprotection [28]; this is also advantageous in AD, which is linked with cardiovascular disease [29].

NE increases cognition/memory through cAMP and ERK signaling [30] and translational regulation for enduring forms of synaptic plasticity called “metaplasticity” [31]. Previously, we reported that CCF219B increased the efficacy but not the potency of NE-mediated cAMP signaling while not affecting the inositol phosphate pathway that regulates vasoconstriction [11]. This bias signaling, a common trait in allosteric modulators, is postulated to increase cognitive and memory functions of CCF219B without affecting blood pressure, a common side effect of α_1A_-AR activators. CCF219B potentiated NE-mediated p-ERK (Figure 1) in transfected α_1A_-AR Rat-1 fibroblasts, demonstrating similar efficacy and potency to the potentiated NE-mediated cAMP signaling [11]. While the Gq-mediated IP/PKC/calcium response is the canonical signaling pathway for α_1A_-AR, it is also thought to activate p38, while ERK signaling by α_1A_-AR has been suggested to be PKC-independent but β-arrestin 2-dependent in transfected HEK cells [18]. Many allosteric modulators in Class A GPCRs have been reported to modulate the receptor through β-arrestin [32]. Interestingly, increases in p-ERK or cAMP have pro-cognitive, memory, and neuroprotective profiles in AD mouse models [14,15,16,17], consistent with CCF219B therapeutic effects [12]. Well known for its role in embryonic development in adults, ERK plays a role in long-term potentiation and emotional and behavioral memory processes, but in neurodegenerative diseases it promotes protection by enhancing synaptic plasticity, decreasing apoptosis, and regulating the inflammatory process [16,33].

Allosteric modulators commonly affect binding kinetics and are considered a sensitive assay for exploring conformational changes. While the dissociation rate commonly decreases with many PAMs, increasing an agonist’s affinity [34], we previously reported that CCF219B increases efficacy without changing affinity [11]. Confirming this, we did not detect changes in ^3^H-NE dissociation when CCF219B was applied in the presence or absence of GTP. While the effect of CCF219B alone also did not appear to change the association rate of ^3^H-NE, it did lower the association rate of ^3^H-NE in the presence of GTP (Figure 2A), suggesting that CCF219B increases a conformation associated with the active state. The conformation induced by CCF219B is likely different than the one induced by GTP alone, as the association rate reduction was synergistic by an order of magnitude. Our results are consistent with those in [35], where GPCR agonists stabilized an active conformation that restricted ligand access and association by adopting a “closed binding site”, resulting in a reduction in the association rate. Our results are also consistent with CCF219B increasing the “pre-active” conformation without changing NE affinity and may allow the receptor to couple more efficiently to the G-protein or other transducer. If CCF219B altered the affinity of NE, changes in the association rate would be apparent without the addition of GTP. The “pre-active” or “pre-coupled activation intermediate” [36] conformation is distinct from the active state and associated with higher ligand efficacy [37]. We are unaware of any previous reports of a GPCR PAM decreasing the association rate and/or in the presence of GTP, as many PAMs increase the affinity of agonists. However, these kinetic results are consistent with our initial findings that CCF219B potentiates the NE-mediated binding and signaling by increasing efficacy without any changes in affinity or potency [11].

We previously explored the structure–activity relationships of our lead compound, CCF219B, and synthesized derivatives with changes in the ortho- or para-substituent groups off the aromatic ring to understand what structural features impart positive allosteric modulation [11]. Extending the length of the aliphatic chain that contains hydrophobic substitutions at the ortho-position is key to its allosteric effects [11]. As the structure of the α_1A_-AR-positive allosteric site revealed extracellular regions distinct from the buried orthosteric agonist site [38] but similar to other PAMs in class A GPCRs [39], we speculate that the ortho-substituted pharmacophore (i.e., 2-yl)methoxy)-2-isobutylphenyl)-), which is longer and more hydrophobic than traditional orthosteric agonists, would bind in this allosteric pocket and effectively slow down the association rate of NE.

CCF219B also induced changes in the equilibrium binding of ^125^I-HEAT, resulting in a small but observable reduction in affinity (Figure 3B). In contrast, for the agonist NE, the association rate of ^125^I-HEAT slightly increased with CCF219B (Figure S2), while the dissociation rate of ^125^I-HEAT appeared to slightly increase (Figure 2D) in the transfected α_1A_-AR membranes. These two rate changes are consistent with a slight reduction in the affinity of ^125^I-HEAT by CCF219B, as assessed by equilibrium binding (Figure 2B). However, we observed much larger increases in the association rate of ^125^I-HEAT when performed in WT mouse brain membranes (Figure 2E). As ^125^I-HEAT is an antagonist, one might expect a different kinetic result compared to agonists, and probe dependence is commonly seen in allosteric modulators. However, our results also suggest that the cellular environment may also play a role, as the allosteric effects were more prominent in endogenous tissue.

While we previously characterized CCF219B using stably transfected Rat-1 fibroblasts of the three α_1_-AR subtypes [11] to demonstrate specificity, we have never performed pharmacological analysis in tissues where conditions are present for endogenous receptor-G-protein levels/interactions, mixed populations of α_1_-AR subtypes, and coupling to other potential signal transducers. As CCF219B first proposed therapeutic use was in AD, we assessed the ex vivo distribution of α_1_-AR subtypes in the normal and AD mouse brain. WT brain had the expected mixed distribution of the subtypes as evidenced by the two-site binding model and consistency with prior binding studies in mouse brain (40–50% high affinity α_1A_-AR) [24,40]. However, we now demonstrate very low α_1A_-AR levels in the brains of the hAPP-Tg AD mice at 9 months of age, compared to age-matched WT (Figure 3A), which are rescued upon preincubation with CCF219B (Figure 6). As CCF219B was removed from the membranes before the binding analysis was performed, the increased in binding sites is not due to an allosteric interaction with ^125^I-HEAT, but some cellular mechanism to increase α_1A_-AR density. In addition, in Figure 2C, CCF219B does not affect the Bmax of ^125^I-HEAT in vitro. hAPP-Tg AD mice have documented deficits in cognitive and memory functions, which CCF219B also rescued in vivo [12]. The α_1A_-AR subtype is highly expressed in the human and rodent hippocampus [41,42], a major center of learning and memory that is affected in AD. The expression of the α_1A_-AR mRNA subtype is also significantly reduced in specific layers of the prefrontal cortex in human subjects with dementia [9]. α_1A_-AR polymorphisms resulting in decreased receptor expression levels are highly linked with schizophrenia [43], while increased receptor expression improved cognition in animal models [8,44]. α_1A_-AR knockout mouse models also display significant loss of learning and memory [8]. Together, these results suggests that AD can be associated with abnormally low levels of the α_1A_-AR. Although it is unknown if this is etiological, it does suggest a potential therapeutic pathway.

While the amount of the α_1A_-AR subtype is decreased in the hAPP-Tg mice, the overall amount of α_1_-ARs are increased. We presumed that this may be part of a compensatory mechanism of α_1A_-AR downregulation with increases in the α_1B_ and/or α_1D_-AR subtype expression. Previous studies indicate that increasing the expression of one α_1_-AR subtype usually downregulates another. Prolonged stimulation by NE increases the expression of α_1A_-AR and downregulates α_1B_ and α_1D_-AR expression in cardiac myocytes [45]. Likewise, transgenic overexpression of the α_1B_-AR leads to a decrease in α_1A_-AR density in the mouse heart [46]. In fact, chronic stimulation and/or expression of the α_1B_-AR in mouse models is associated with neuropathophysiology. Overexpression of the α_1B_-AR has led to seizures [47], neuronal death [48,49], synucleinopathy [50], neurodegeneration [51], decreased lifespan [52], while α_1B_-AR knock out confers resistance to neurotoxicity and seizures [53], and increased lifespan [52]. However, a new property of CCF219B appears to increase or stabilize the expression of α_1A_-AR in a positive feedback loop. We confirmed this by preincubating CCF219B with brain membranes from the hAPP-Tg mice for 4–5 h which increased ^125^I-HEAT-binding sites and the high affinity α_1A_-AR proportion (Figure 6). We speculate that as increased NE stimulation upregulates α_1A_-AR [45], perhaps the loss of NE neurons in the locus coeruleus, a prominent pathophysiology in AD [54], results in decreased α_1A_-AR expression. In corroboration, previous studies in the prefrontal cortex of dementia patients indicated increased postsynaptic expression of the α_1_-ARs but a specific downregulation of α_1A_-AR mRNA [9]. Our results suggest that the downregulation of α_1A_-AR and the compensatory increased expression of the α_1B_-AR subtype may play a role in AD progression. In addition, CCF219B potency to reverse AD symptoms may also be due, in part, to a positive feedback loop that increases α_1A_-AR expression and its resultant signaling pathways.

The mechanism of the increased α_1A_-AR expression remains to be determined but it is not due to increased protein synthesis as cycloheximide had no effect. Increased surface expression can also occur from recycling of internalized proteins, increased protein stability, or decreased catabolism. Elevating temperature generally decreases protein stability. While interactions of receptors with their ligands can often increase protein thermostability [55], increased temperature also results in an decrease in affinity for agonists [20]. However, we did not see any changes in protein expression or potency when CCF219B binding was conducted at 37 °C, suggesting that CCF219B may be contributing to increased protein/complex stability. As with the association studies of ^125^I-HEAT, CCF219B has more prominent allosteric effects on receptor expression when conducted in endogenous tissues. As the transfected cell line expresses approximately 1000-fold more receptor than in endogenous settings, there is a dominance of uncoupled receptors which may limit CCF219B’s allosteric effects that are associated with pre-coupled receptors. There are also many examples for altered receptor surface expression in the literature with allosteric modulators upon prolonged exposure [56,57,58], which suggest their ability to alter the internalization and/or trafficking of normal or misfolded receptors [59,60].

CCF219B is currently in pre-investigational new drug (IND)-enabling studies and successfully completed 14-day repeated dosing studies in both rats and dogs without any major adverse reactions, including changes in blood pressure. Our hope is that CCF219B would bring clinical relevance in the long-term relief of cognitive deficits to AD patients without significant side effects.

## 4. Materials and Methods

### 4.1. Animals

All animal studies were performed according to the NIH Guide for the Care and Use of Laboratory Animals and experimental protocols were reviewed by the Cleveland Clinic animal care and use committee (protocol #00002733, approval 7 October 2024). For radioligand-binding studies, brains from both hAPP[V717I] transgenic mice (FVB/N x C57Bl/6J background; hAPP-Tg(lon)) were obtained through the CRO ReMYND (Leuven-Heverlee, Belgium). The WT controls were non-transgenic littermates with the same genetic background. The age at sacrifice was 8.3 or 8.4 months old.

### 4.2. Compounds

CCF219B (previously referred to as Cmpd-3; *N*-(3-((4,5-Dihydro-1*H*-imidazol-2-yl)methoxy)-2-isobutylphenyl)-*N*-methylmethanesulfonamide hydrochloride) was synthesized by Medicilon (Shanghai, China), verified by LC-MS and NMR to be 98^+^% purity, and is highly soluble in water. Details on the structure, synthesis, and characterization have been published [11].

### 4.3. MAPK Assays

Rat-1 fibroblasts stably expressing α_1A_-AR were cultured as previously described [11]. Cells were re-plated into 6-well dishes in Dulbecco’s Modified Eagle Medium (DMEM) (Sigma-Aldrich, Burlington, MA, USA) media without serum at approximately 2 × 10^6^ cells/well. Cells were allowed to rest till the next day in a CO_2_ incubator at 37 °C. The cells were washed twice with DMEM (no serum); then, 2 mL of DMEM was added to each well. Cells are pre-incubated for 1 h at 37 °C with β- and α_2_-AR blockers, 10 μM propranolol, and 1 μM rauwolscine. Time-course studies of MAPK activation were first performed using either norepinephrine (NE) alone (10^−4^ M) or in the presence of CCF219B (10^−8^ M). Thereafter, cells are incubated for 20 min with either NE alone (10^−4^ M) or in the presence of CCF219B at various doses (10^−14^ through 10^−5^ M), the reaction stopped using cold lysis buffer, and the samples stored at −20 °C. The samples were processed for total protein determination and SDS electrophoresis using 10% Tris gels and then transferred to nitrocellulose, as previously described [11]. The nitrocellulose was blocked with 5% BSA in Tris-buffered saline with Tween 20 (TBST) for 1 h at room temperature, washed 3 × 5 min with TBST, and then incubated overnight at 4 °C with rabbit phospho- or unphosphorylated p44/42 antibody (Cell Signaling, Danvers, MA, USA; #9101 or #9102) or rabbit phospho- or unphosphorylated p38 antibody (Cell Signaling #9211 or #9212) or GAPDH (Cell Signaling #2118) in 5% BSA in TBST at 1:1000 dilution. The membrane was washed 3 × 5 min before incubating with 1:10,000 goat anti-rabbit IgG HRP in 3% milk in TBST for 1 h at room temperature. After washing 5 × 5 min, the membrane was incubated for 1 min with 4 mL of each component of the Pierce SuperSignal Chemiluminescent Substrate kits (Thermo Fisher Scientific, Waltham, MA, USA) and exposed to X-ray film (Cl-Xposure). The bands were quantified using the Image Studio Digits Version 4.0 program associated with the Li-Cor digital scanner model C-DIGit (LiCORbio, Lincoln, NE, USA)and graphed using GraphPad Prism (San Diego, CA, USA) software (version 10) and corrected for loading using GAPDH or total ERK.

### 4.4. Mouse Brain Membrane Preparation

Frozen brain samples were diced and transferred into 10 mL of ice-cold buffer A (10 mM HEPES, pH 7.4, 250 mM sucrose, 5 mM EGTA, 12.5 mM MgCl_2_) containing the following protease inhibitors (20 μg/mL aprotinin, 20 μg/mL leupeptin, 20 μg/mL bacitracin, 20 μg/mL benzamide, 17 μg/mL PMSF, and 1× of Calbiochem protease inhibitor cocktail (Cat# 539134). Tissue was homogenized for 30 s in a Brickmann Instruments (Riverview, FL, USA) PT3000 polytron at 29,000 rpm on ice. The sample was then transferred to a dounce homogenizer and dounced 10 times with a loose pestle, followed by 10 dounces with a tight pestle. The sample was then transferred to a ground glass homogenizer and dounced an additional 10 times. The sample was centrifuged for 5 min at 209× *g* at 4 °C to remove nuclei and large particles. The supernatant was then centrifuged at 23,000× *g* at 4 °C for 15 min. The pellet was resuspended in ice-cold buffer B (10 mM HEPES, pH 7.4, 100 mM NaCl, 5 mM EGTA, 12.5 mM MgCl_2_) containing the same cocktail of protease inhibitors and recentrifuged at 23,000× *g* at 4 °C for 15 min. Pellets were resuspended in ice-cold Buffer B containing 10% glycerol and homogenized with a Teflon pestle, aliquoted, and stored at −70 °C.

### 4.5. Radioligand-Binding Studies

Binding assays were performed in duplicate in HEM buffer (20 mM HEPES, pH 7.4, 1.4 mM EGTA, 12.5 mM MgCl_2_, pH to 7.4) supplemented with 0.005% BSA, 10 μM propranolol, and 1 μM rauwolscine, in a total assay volume of 1 mL. It was composed of 10–100 μg of brain membranes or membranes prepared from α_1A_-AR stably transfected Rat-1 fibroblasts titrated with various concentrations of CCF219B. Binding components were incubated for various timepoints at 25 °C with 240 pM or increasing ^125^I-(2-{[β-(4-Hydroxyphenyl) ethyl]aminomethyl}-1-tetralone hydrochloride) (HEAT) concentrations before cell harvesting (Brandel, Gaithersburg, MD) through Whatman (Buckinghamshire, UK) GF/C filters treated with 0.33% polyethylenimine. Filters were washed rapidly with 20 mL of ice-cold HEM buffer to remove further nonspecifically bound radioactivity. Non-specific binding is determined using 100 μM phentolamine. Binding data were analyzed using GraphPad Prism, version 10.

Association binding experiments with or without 5 mM GTP were performed either at room temperature or in an ice-bath using membranes from Rat-1 fibroblasts transfected with α_1A_-AR or membranes prepared from WT mouse brains and the same buffer and blockers used in the equilibrium binding experiments. A total of 0.018% ascorbic acid was also added to the buffers to inhibit oxidation for experiments using the agonist ^3^H-NE. Dissociation experiments were measured in the presence of saturating amounts of either unlabeled NE or EPI (10^−3^ M) to prevent the re-association of ^3^H-NE. Because of the fast kinetics, a 12-well sampling vacuum filtration manifold was used (Millipore XX270550, Merk Millipore, Burlington, MA, USA) as the cell harvester. Data were analyzed using GraphPad Prism with one-phase decay for association rates and a two-phase decay for dissociation.

In pre-incubation studies, brain membranes from the hAPP-Tg mice were pre-incubated with CCF219B (10^−8^ M) or with HEM buffer for 4–5 h at 25 °C in a shaking water bath. The membranes were washed three times with HEM by centrifugation, and the supernatant was removed to eliminate all traces of CCF219B. The membranes were resuspended in HEM and re-dounced with a Teflon homogenizer. Competition binding studies were performed using ^125^I-HEAT and various concentrations of phentolamine for 1 h at 25 °C to determine the amount of high- and low-affinity sites.

### 4.6. Statistical Analysis

Statistical testing was performed using an ANOVA and Tukey’s post hoc multiple comparison test to determine significant differences or a student’s *t*-test. Some binding curves, particularly those showing weak allosteric effects, were best-fitted using GraphPad Prism to go through all data points, using either a spline or bell shape, where x is the concentration and analyzed for significance using the Mann–Whitney test. Inhibition curves were best-fitted to either a one-site or two-site log IC_50_ model using the standard equations in GraphPad Prism. Data are presented as the mean. The error is reported as the standard error of the mean. Significance was determined at *p* < 0.05.

## 5. Conclusions

In summary, CCF219B can have multiple effects on ligand binding which are both ligand- and cellular-dependent. CCF219B appears to alter only the association binding kinetics of NE, in contrast with other GPCR PAMs, which effect the dissociation rate, but consistent with enhancing the formation of a pre-coupled activation intermediate, allowing for more efficient coupling and allosteric effects on increasing efficacy. Potentiation of pERK may contribute to CCF219B’s ability, along with cAMP, to confer cognitive, memory, and neuroprotective benefits in AD. The ability of CCF219B to increase the expression of its own receptor may be important in AD, where the expression of α_1A_-AR is downregulated. This also implies that the effects of CCF219B would be long-lasting and would not undergo desensitization of the receptor and its downstream signaling, a common feature of neurotransmitter modulation.

## Data Availability

The data presented in this study are available upon reasonable request from the corresponding author to protect potential patentable aspects of the research.

## Supplementary Materials

The following supporting information can be downloaded at: https://www.mdpi.com/article/10.3390/ph18040476/s1, Figure S1: MAPK time-course studies; Figure S2: Association curves of ^125^I-HEAT (40 pM) with or without CCF219B (10^−5^ M) in stably transfected α_1A_-AR membranes; Figure S3: Prolonged incubation of CCF219B in stably transfected α_1A_-AR membranes masks the high affinity site.
