# Supplementary figures and images for: Further In Vitro and Ex Vivo Pharmacological and Kinetic Characterizations of CCF219B: A Positive Allosteric Modulator of the α1A-Adrenergic Receptor
Source: Pharmaceuticals (Basel). 2025 Mar 27;18(4):476. doi: 10.3390/ph18040476 (PMC12030198; doi:10.3390/ph18040476)

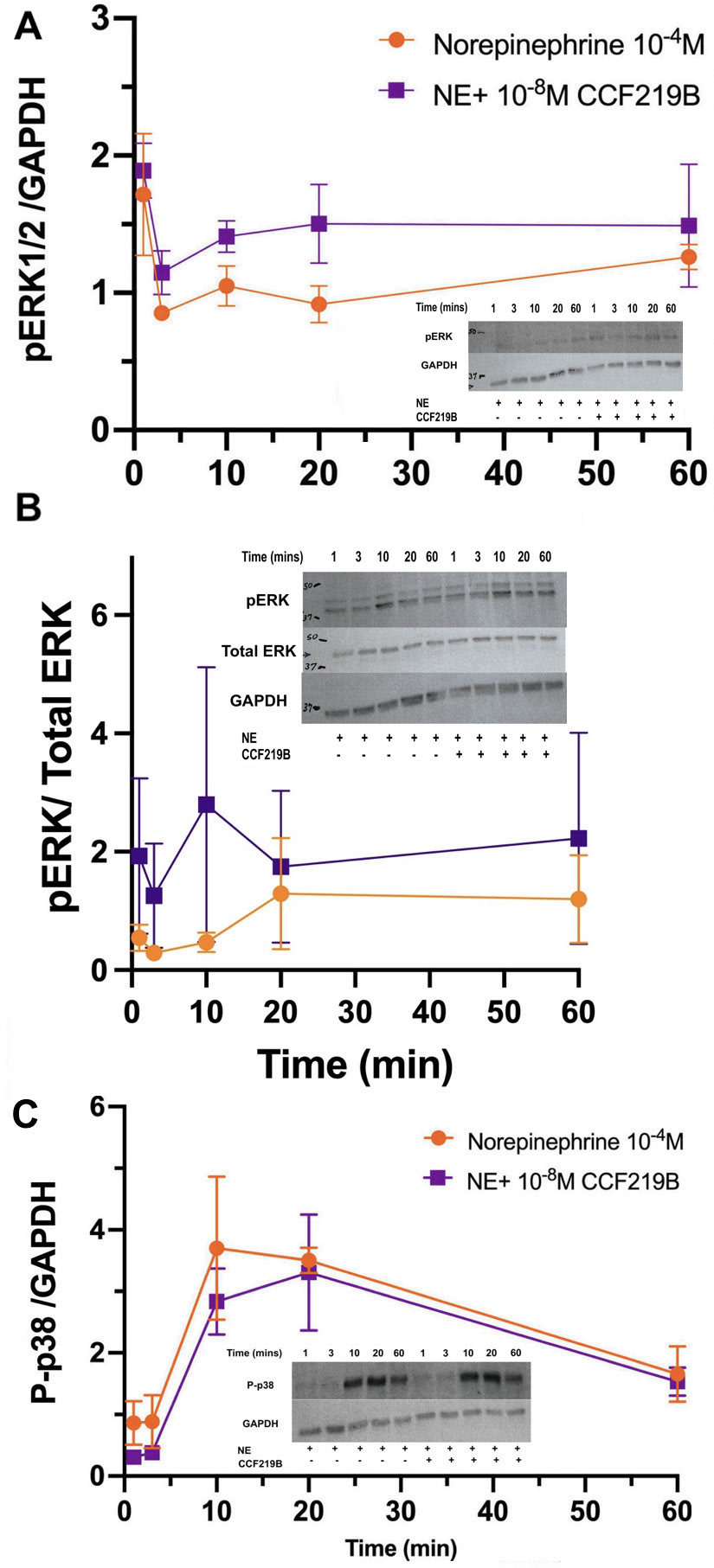

Supplement: Supplementary file 1 [file pharmaceuticals-18-00476-s001.zip › Fig. S1ABC pERK:GAPDH revised copy 2.jpg]

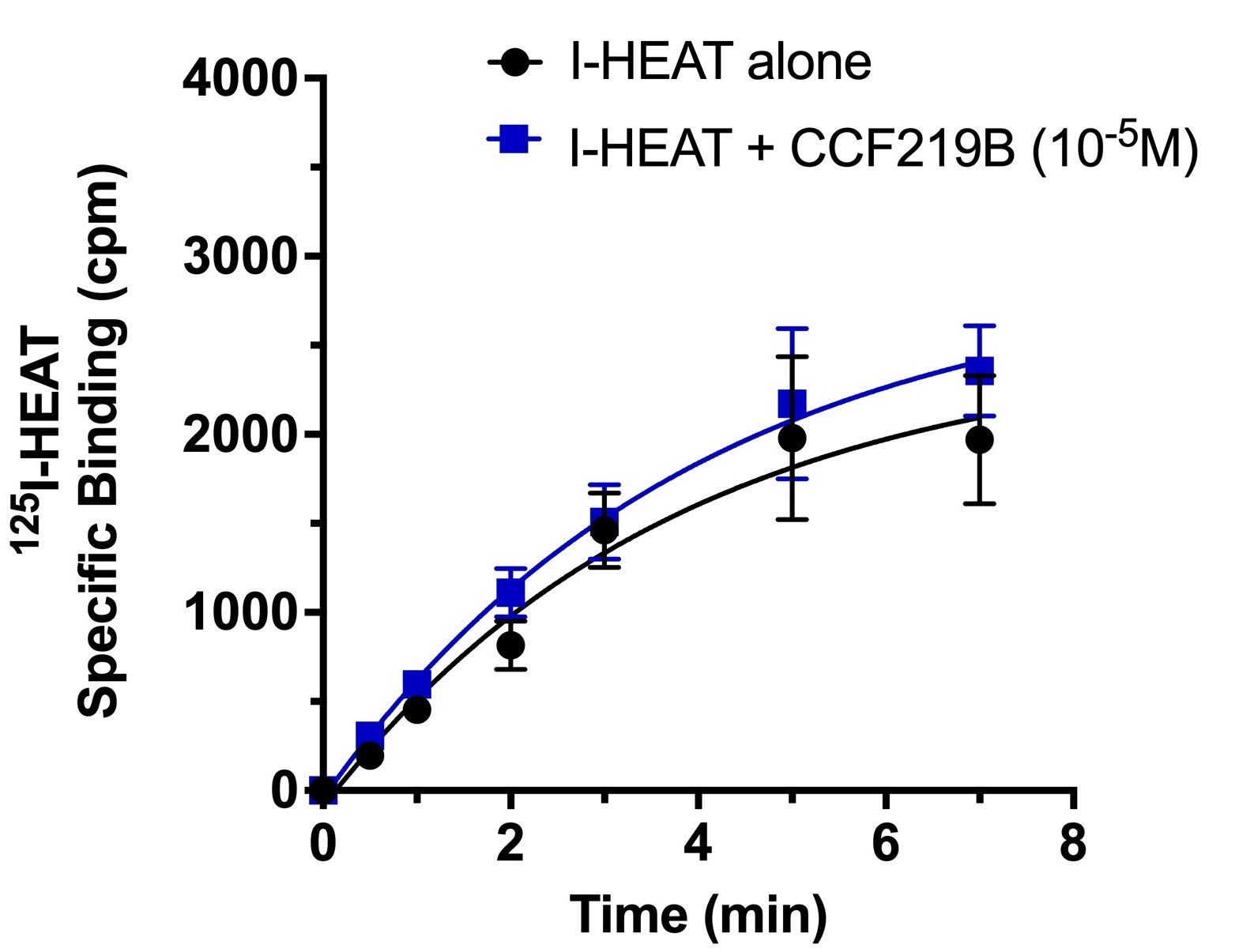

Supplement: Supplementary file 1 [file pharmaceuticals-18-00476-s001.zip › Fig. S2 IHEAT association rat 1 membranes copy 2.jpg]

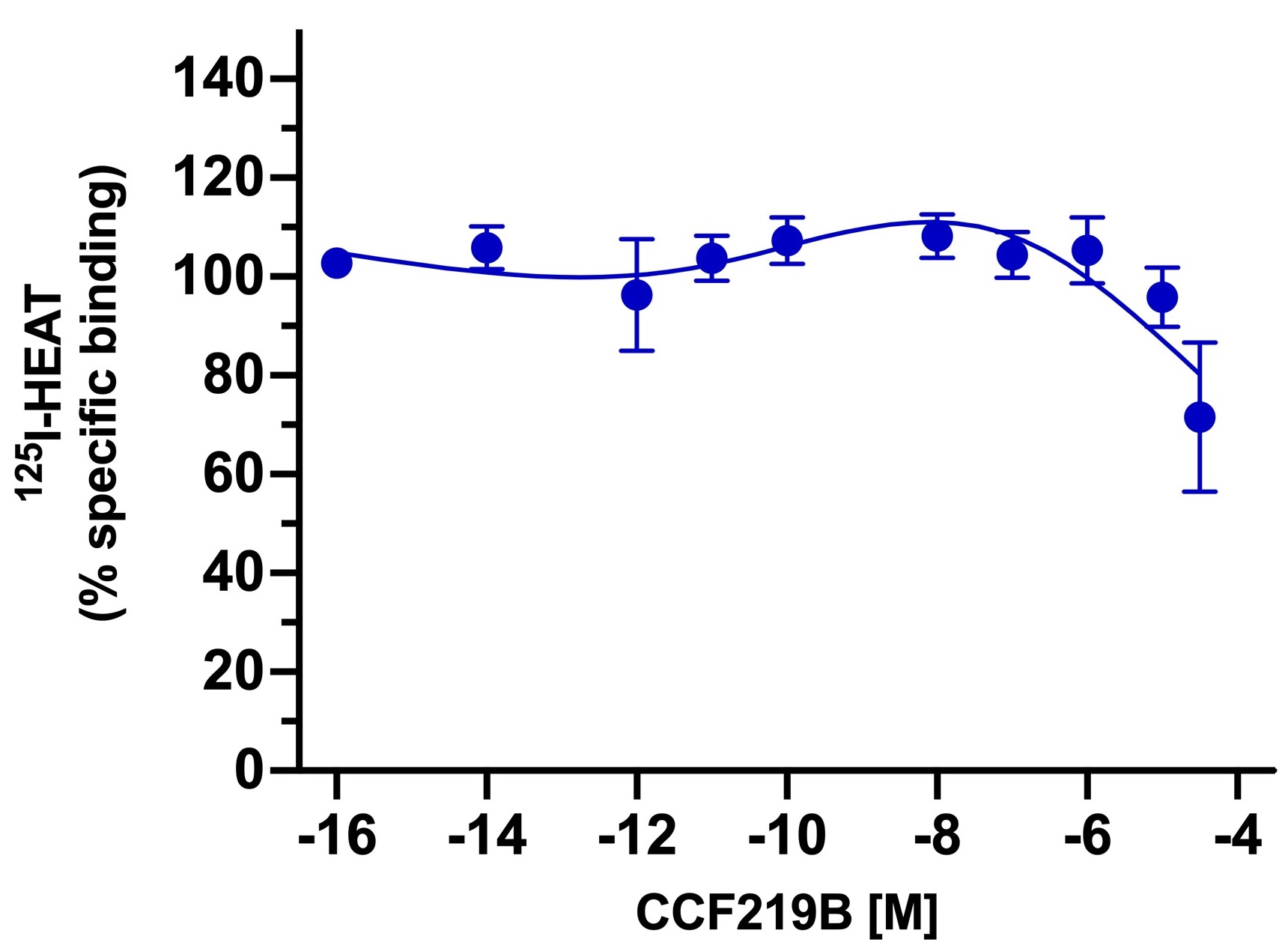

Supplement: Supplementary file 1 [file pharmaceuticals-18-00476-s001.zip › Fig.S3 revised copy.jpg]
